# A Rapid Immunochromatography Test Based on Hcp1 Is a Potential Point-of-Care Test for Serological Diagnosis of Melioidosis

**DOI:** 10.1128/JCM.00346-18

**Published:** 2018-07-26

**Authors:** Phornpun Phokrai, Wisansanee Karoonboonyanan, Nida Thanapattarapairoj, Chidchanok Promkong, Adul Dulsuk, Sirikamon Koosakulnirand, Sasha Canovali, Nitaya Indrawattana, Yaowaruk Jutrakul, Vanaporn Wuthiekanun, Direk Limmathurotsakul, Paul J. Brett, Mary N. Burtnick, Ganjana Lertmemongkolchai, Narisara Chantratita

**Affiliations:** a Department of Microbiology and Immunology, Faculty of Tropical Medicine, Mahidol University, Bangkok, Thailand; b Medical Technology Department, Khon Kaen Hospital, Khon Kaen, Thailand; c Medical Technology Department, Udon Thani Hospital, Udon Thani, Thailand; d Department of Medical Laboratory, Nakhon Phanom Hospital, Nakhon Phanom, Thailand; e Mahidol-Oxford Tropical Medicine Research Unit, Mahidol University, Bangkok, Thailand; f Department of Tropical Medicine, Medical Microbiology and Pharmacology, John A. Burns School of Medicine, University of Hawaii at Manoa, Honolulu, Hawaii, USA; g Department of Tropical Hygiene, Faculty of Tropical Medicine, Mahidol University, Bangkok, Thailand; h Department of Microbiology and Immunology, University of Nevada, Reno School of Medicine, Reno, Nevada, USA; i The Cellular and Molecular Immunology Unit, The Center for Research and Development of Medical Diagnostic Laboratories (CMDL), Faculty of Associated Medical Sciences, Khon Kaen University, Khon Kaen, Thailand; University of Tennessee at Knoxville

**Keywords:** Burkholderia pseudomallei, immunochromatography test, antibody detection, melioidosis, point-of-care test, rapid test, serological diagnosis

## Abstract

Melioidosis is a fatal infectious disease caused by the environmental bacterium Burkholderia pseudomallei. It is highly endemic in Asia and northern Australia but neglected in many other tropical countries. Melioidosis patients have a wide range of clinical manifestations, and definitive diagnosis requires bacterial culture, which can be time-consuming. A reliable rapid serological tool is greatly needed for disease surveillance and diagnosis. We previously demonstrated by enzyme-linked immunosorbent assay (ELISA) that a hemolysin-coregulated protein (Hcp1) is a promising target for serodiagnosis of melioidosis. In this study, we developed a rapid immunochromatography test (ICT) using Hcp1 as the target antigen (Hcp1-ICT). We evaluated this test for specific antibody detection using serum samples obtained from 4 groups of human subjects, including the following: (i) 487 culture-confirmed melioidosis patients from four hospitals in northeast Thailand; (ii) 202 healthy donors from northeast Thailand; (iii) 90 U.S. healthy donors; and (iv) 207 patients infected with other organisms. Compared to culture results as a gold standard, the sensitivity of ICT for all hospitals was 88.3%. The specificities for Thai donors and U.S. donors were 86.1% and 100%, respectively, and the specificity for other infections was 91.8%. The results of the Hcp1-ICT demonstrated 92.4% agreement with the Hcp1-ELISA results with a kappa value of 0.829, indicating that the method is much improved compared with the current serological method, the indirect hemagglutination assay (IHA) (69.5% sensitivity and 67.6% specificity for Thais). The Hcp1-ICT represents a potential point-of-care (POC) test and may be used to replace the IHA for screening of melioidosis in hospitals as well as in resource-limited areas.

## Abstract (precis)

Melioidosis is a fatal infectious disease caused by the environmental bacterium Burkholderia pseudomallei. It is highly endemic in Asia and northern Australia but neglected in many other tropical countries.

## INTRODUCTION

Melioidosis is an infection caused by Burkholderia pseudomallei. This bacterium is a Gram-negative bacillus that is ubiquitous in soil and water in tropical and subtropical regions (1). B. pseudomallei can survive in extreme environments and can infect both humans and animals (1). The global burden of the disease is substantial and growing due to widespread dissemination of B. pseudomallei and increases in populations with predisposing risk factors (1, 2). Risk factors for melioidosis include diabetes mellitus, chronic kidney disease, excessive alcohol consumption, exposure to soil and water in regions of endemicity, male sex, age of >45 years, liver disease, lung disease, thalassemia, prolonged steroid use, and immunosuppression (1). Skin inoculation is the main route of infection, but inhalation during severe weather events and ingestion of contaminated water can also lead to B. pseudomallei infections (3). Due to diagnostic limitations and underrecognition, melioidosis is believed to be underreported in many tropical countries where the disease is endemic or predicted to be endemic (4).

Melioidosis is estimated to affect 165,000 people worldwide, mainly in Asia and Northern Australia, causing 89,000 deaths annually (4). The disease is reported to be the third leading cause of death from infectious disease in Thailand after HIV and tuberculosis (5). It is estimated that Thailand has the largest number of melioidosis cases in the region, with 2,000 to 3,000 cases occurring each year (4). The disease is known as the great mimicker, since the clinical symptoms of patients are similar to those seen with other diseases and range from skin abscesses to acute pneumonia and septicemia (6). Laboratory diagnosis is currently limited in some areas of endemicity, contributing to mortality rates as high as 50% in some developing countries. In contrast, mortality rates of 10% are observed in the more developed areas that have access to advanced intensive care therapy for severe sepsis (1). The mortality rate of the disease in Thailand is approximately 40% but can reach as high as 90% in cases of severe sepsis (3). Early diagnosis is critical since standard therapies used to treat sepsis in areas of endemicity are often ineffective for B. pseudomallei. Treatment of melioidosis requires intravenous ceftazidime or meropenem for at least 10 to 14 days followed by 3 to 6 months of oral treatment with trimethoprim-sulfamethoxazole (3). The response to antibiotic treatment can be slow, with a median time to resolution of fever of up to 9 days (1). In order to effectively treat infected patients, rapid and accurate diagnosis is essential.

The current gold standard for identifying B. pseudomallei infections involves isolating the bacteria from clinical specimens such as blood, urine, sputum, fluid from abscesses, and throat swabs and further identification by standard biochemical tests, latex agglutination (7, 8), or matrix-assisted laser ionization–time of flight (MALDI-TOF) (9). The culture method often takes from 2 to 7 days, with only 60% sensitivity (10). Culturing B. pseudomallei requires experience and strict laboratory safety procedures; further complicating diagnosis, B. pseudomallei is often be misidentified as Pseudomonas species (11). The laboratory facilities required are not available in primary hospitals in Thailand and other areas of endemicity; as a result, a large number of B. pseudomallei-infected patients in rural areas are believed to be undiagnosed. The use of quantitative real-time PCR can generate results more quickly than culture but has low (61%) sensitivity when used in Thailand (12). Other tests are available, such as latex agglutination and immunofluorescence assays, but all present similar problems, with an increased time to culture required prior to testing or low sensitivity or no availability of the instruments needed for these types of tests. A lateral flow immunoassay (LFI) that detects a capsular polysaccharide has been developed (13), and although it shows good specificity, it has been reported to have poor sensitivity for blood samples (14).

Serological diagnosis can improve the speed of diagnosis. An indirect hemagglutination assay (IHA) has been widely used to determine the antibody titers that are indicative of exposure to B. pseudomallei, but many studies have shown that the sensitivity and specificity of this test are low in regions of endemicity (15–17). Recently, we developed rapid enzyme-linked immunosorbent assays (ELISAs) using O-polysaccharide (OPS) and hemolysin-coregulated protein 1 (Hcp1) and demonstrated that these antigens are promising candidates for the serodiagnosis of melioidosis (18, 19). Hcp1 is a component of a virulence-associated type VI secretion system (T6SS) that plays a role in the intracellular lifestyle of B. pseudomallei (20). Our previous study showed that the Hcp1-ELISA performed better than the OPS-ELISA in evaluations in areas of endemicity and suggested that Hcp1 represents a promising target antigen for the development of a point-of-care (POC) test for melioidosis diagnosis (19).

A simple and rapid diagnosis method is greatly needed for melioidosis. Here, we have developed an immunochromatography test (ICT) based on the Hcp1 antigen (Hcp1-ICT) which can detect antibody in serum samples in 15 min. We evaluated the Hcp1-ICT using different sets of serum samples from 487 patients with culture-confirmed melioidosis in 4 provinces in northeast Thailand, 207 Thai patients with other bacterial infections, 202 healthy donors in northeast Thailand, and 90 healthy donors in the United States. We compared the results of the Hcp1-ICT with the results of the Hcp1-ELISA and IHA. Our study data demonstrate that the Hcp1-ICT is a promising POC test for serological diagnosis of melioidosis.

## MATERIALS AND METHODS

### Ethical approval.

This study was approved by the Human Research Ethic Committee of the Faculty of Tropical Medicine, Mahidol University (approval numbers MUTM 2012-018, MUTM 2014-079, and MUTM 2016-075); Udon Thani Hospital (approval number 2/2560); Khon Kaen Hospital (approval numbers KE600 and 18); and Nakhon Phanom Hospital (approval number NP-EC11-2/2560).

### Serum samples.

The human serum samples used to develop and evaluate the Hcp1-ICT were anonymous and included the following sets: (i) 487 on-admission sera from culture-confirmed melioidosis patients who were admitted to Sunpasitthiprasong Hospital, Ubon Ratchathani (*n* = 141), Udon Thani Hospital, Udon Thani (*n* = 198), Khon Kaen Hospital, Khon Kaen (*n* = 91), and Nakhon Phanom Hospital, Nakhon Phanom (*n* = 57) in northeast Thailand; (ii) 202 sera from healthy donors in Ubon Ratchathani (*n* = 188) and healthy donors in Udon Thani (*n* = 14) in northeast Thailand; (iii) sera from healthy donors in the United States (*n* = 90) (Innovative Research, Novi, MI, USA); and (iv) sera from patients with culture results of other bacterial or fungal infections (*n* = 207). These serum samples were obtained from patients at Udon Thani Hospital (*n* = 50), Khon Kaen Hospital (*n* = 154), and Nakhon Phanom Hospital (*n* = 3) in northeast Thailand. The other organisms isolated from blood or other specimens of patients were as follows: Acinetobacter baumannii (*n* = 21), Aeromonas spp. (*n* = 1), Bacillus spp. (*n* = 4), Burkholderia cepacia (*n* = 1), Citrobacter koseri (*n* = 2), coagulase-negative staphylococci (*n* = 20), Corynebacterium spp. (*n* = 5), Cryptococcus neoformans (*n* = 2), Candida albicans (*n* = 9), Candida glabrata (*n* = 1), Candida spp. (*n* = 1), Clostridium perfringens (*n* = 1), Enterococcus faecium (*n* = 3), Escherichia coli (*n* = 15), Elizabethkingia meningoseptica (*n* = 1), Enterobacter aerogenes (*n* = 1), Enterococcus faecalis (*n* = 1), Enterococcus spp. (*n* = 5), Haemophilus influenzae (*n* = 1), Klebsiella pneumoniae (*n* = 13), K. oxytoca (*n* = 1), Leptospira interrogans (*n* = 20), Micrococcus spp. (*n* = 2), Morganella morganii (*n* = 1), Orientia tsutsugamushi (*n* = 20), Pseudomonas aeruginosa (*n* = 3), Pseudomonas spp. (*n* = 1), Streptococcus agalactiae (*n* = 8), S. pneumoniae (*n* = 2), S. anginosus (*n* = 1), S. dysgalactiae (*n* = 1), S. gordonii (*n* = 1), S. parasanguinis (*n* = 1), S. pyogenes (*n* = 2), group B streptococcus (*n* = 1), non-group A, B, or D streptococci (*n* = 1), Staphylococcus aureus (*n* = 15), viridans streptococci (*n* = 2), and organisms representing polymicrobial infections (*n* = 15).

### Development of Hcp1-ICT for antibody detection.

The rapid ICT was developed using a recombinant Hcp1 (rHcp1) antigen (19). The optimal concentration of antigen was initially determined by using pooled melioidosis and pooled healthy sera (5 patients with culture-confirmed melioidosis and 5 healthy Thai donors, respectively). The antigen was diluted in phosphate-buffered saline (PBS) buffer (pH 7.4) at a dilution range of 0.5, 1, and 2 mg/ml. The optimal concentration of serum dilution was defined by using 2-fold serial dilution of the pooled melioidosis sera and pooled healthy sera at a dilution range of 1:10 to 1:2,000. Each antigen was sprayed onto a UniSart CN 140 membrane (Sartorius) using an IsoFlow reagent dispenser and dried in an oven (Binder, Germany). The conjugate pad was sprayed with colloidal gold-conjugated mouse antihuman IgG (Kestrel Bioscience, Thailand). The control line was coated with 1 mg/ml goat anti-mouse immunoglobulins (Serve Science, Thailand). The membrane was cut into a 4-mm strip using a Kinematric automachine (Kinematic Automation, USA) and assembled with a sample pad (Whatman), a conjugate pad, and an absorbent pad (Whatman) in a dehumidifier room. The assembly of ICT was performed by Kestrel Bioscience, Thailand. Serum samples in different dilutions were tested. A total of 120 μl of the diluted sera was added to sample wells of the ICT and incubated at room temperature for 15 min.

Following optimization, the strip was prepared using the optimized antigen concentration and kept in a sealed aluminum bag with silica gel at room temperature until use. On the day that the ICT was performed, the strip was removed from the aluminum bag and the reactivity of individual serum samples was determined using the optimized conditions.

### Hcp1-ICT testing.

**FIG 1:**
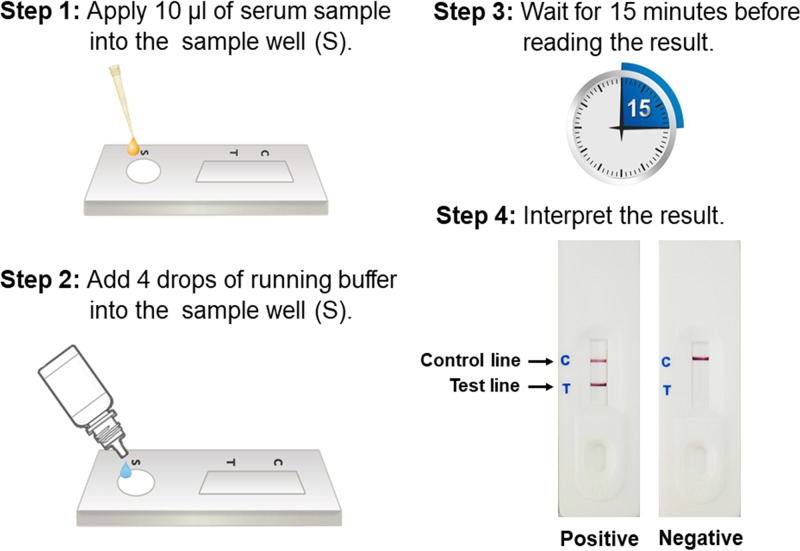
Immunochromatography test (ICT) procedure. A serum sample (10 μl) from a suspected melioidosis patient is applied to the sample well followed by addition of four drops (120 μl) of running buffer to the sample well. The test was allowed to process the sample for 15 min before the result was read. The result was evaluated by the presence of a control line with either a visible or nonvisible test line. The presence of a red line at the test line was interpreted as representing a positive result, while the absence of a red line at the test line was interpreted as representing a negative result.

A 10-μl volume of serum samples was applied to the sample well followed by the addition of 4 drops (120 μl; 30 μl per drop) of running buffer (Fig. 1). Results were read at room temperature after 15 min. A valid positive result was determined by the presence of reddish lines at the test line (T) and control line (C). A negative result was determined by the presence of a reddish line at the control line (C) only.

### Comparison of the Hcp1-ELISA and IHA.

The Hcp1-ELISA was performed using a 1:2,000 dilution of serum as previously described (19). A 1:2,000 dilution of horseradish peroxidase-conjugated rabbit antihuman IgG was used as previously described (19). The IHA was performed as described by Suttisunhakul et al. (15). Positive results for individual serum samples were determined using an optical density (OD) cutoff value of 1.165 for ELISA (19) and an antibody titer of ≥1:160 for IHA (15).

### Comparison between Hcp1-ICT and Hcp1-ELISA.

The Hcp1-ICT was used to detect antibodies to Hcp1 in pooled positive and pooled negative serum samples, and the results were compared with the values for OD at 450 nm (OD_450_) obtained from the Hcp1-ELISA. The serum sample was serially diluted with buffer to yield the desired OD_450_ values of ELISA (OD = 0.05 to 3.50). Each dilution of serum (10 μl) was mixed with 120 μl of ICT running buffer. The mixture was applied to the sample well on the cassette. The Hcp1-ICT test was allowed to develop for 15 min. The OD value of each Hcp1-ELISA reaction that showed positive Hcp1-ICT results was read.

### Evaluation of rapid Hcp1-ICT for diagnosis of melioidosis.

A total of 986 anonymous human serum samples were used to evaluate the Hcp1-ICT for diagnosis of melioidosis. The first set included 141 on-admission sera from culture-confirmed B. pseudomallei-infected patients who were admitted to Sunpasitthiprasong Hospital, Ubon Ratchathani, northeast Thailand; 188 serum samples from healthy donors from the same area in northeast Thailand; and 90 serum samples from U.S. healthy donors. These samples were used to compare the results of Hcp1-ICT and Hcp1-ELISA and IHA. The results of the Hcp1-ELISA and IHA determined using the same set of samples were obtained from our previous study (15, 19). Additional anonymous human sera from 346 patients with culture-confirmed B. pseudomallei infection, 14 Thai healthy donors, and 207 patients with other organisms from three different hospitals in northeast Thailand were further used to evaluate the sensitivity and specificity of the Hcp1-ICT. Evaluation of the Hcp1-ICT was conducted in these hospitals between 2016 and 2017. The hospitals included (i) Udon Thani Hospital, Udon Thani; (ii) Khon Kaen Hospital, Khon Kaen; and (iii) Nakhon Phanom Hospital, Nakhon Phanom. Patients with suspected melioidosis were identified with the requests from clinicians for bacterial culture from any clinical specimen. Hcp1-ICT was performed on leftover serum samples from these on-admission patients by the hospital staff before the culture results were known.

### Statistical analysis.

Statistical analyses were performed using Stata version 12 (StataCorp LP, College Station, TX). The sensitivity and specificity of Hcp1-ICT, Hcp1-ELISA, and IHA were calculated using bacterial culture results as a gold standard. The McNemar test was used to compare the sensitivity and specificity data between the different tests. The agreement between different tests was determined by Kappa analysis. Fisher's exact test was used to test the difference between the Hcp1-ICT results and the diabetic status of patients. Differences were considered statistically significant if the *P* value was ≤0.05.

## RESULTS

### Optimization of Hcp1-ICT for antibody detection.

The optimal conditions for the Hcp1-ICT were initially determined using pooled serum from either melioidosis patients or healthy donors. The optimized concentrations of the coating antigens were determined to be 0.5 mg/ml and 1 mg/ml for Hcp1 antigen on the membrane. The tests were standardized by precoating the ICTs and placing them in sealed aluminum bags with silica gel and storing them dried at room temperature until required for use.

To determine the optimal serum dilution for screening of the serum samples, we tested 2-fold serial dilutions of pooled serum from culture-confirmed melioidosis patients or Thai healthy donors. The greatest differences in reactions with Hcp1 antigen between the melioidosis and healthy donors were obtained at serum dilutions of 1:10 to 1:20. The assay was therefore performed using 10 μl of serum and 4 drops (120 μl) of running buffer from a reagent bottle (at a serum dilution of 1:13) at room temperature (25°C) for 15 min. The assay was standardized throughout the study using this dilution for all serum samples.

### Comparison of Hcp1-ICT, Hcp1-ELISA, and IHA for diagnosis of melioidosis.

To determine the IgG antibody specific to Hcp1, ICTs with 0.5 and 1 mg/ml of Hcp1 antigen were performed with first set of 419 human serum samples from melioidosis patients, Thai healthy donors, and U.S. healthy donors. The results of the Hcp1-ICT assay were compared with those of Hcp1-ELISA and IHA from our previous studies (15, 19) (Table 1). The diagnostic sensitivity and specificity of Hcp1-ICT were not different using Hcp1 at 0.5 and Hcp1 at 1 mg/ml (sensitivity, 89.4% versus 90.1% [*P* = 1.000]; specificity, 86.2% versus 84.0% [*P* = 0.133 for Thai healthy donors] and 100% for both using Hcp1 at 0.5 mg/ml and at 1 mg/ml for U.S. healthy donors).

**TABLE 1:** Sensitivity and specificity of Hcp1-ICT at concentrations of 0.5 mg/ml and 1 mg/ml^*a*^

Assay (cutoff value)	% sensitivity (CI)	% specificity (CI)	
	Thai melioidosis patients (*n* = 141)	Thai healthy donors (*n* = 188)	U.S. healthy donors (*n* = 90)
Hcp1-ICT at 0.5 mg/ml (positive band)	89.4 (83.1–93.9)	86.2 (80.4–90.8)	100 (96.0–100)
Hcp1-ICT at 1.0 mg/ml (positive band)	90.1 (83.9–94.5)	84.0 (78.0–89.0)	100 (96.0–100)
Hcp1-ELISA (OD ≥ 1.165)	83.0 (75.7–88.8)	96.3 (92.5–98.5)	95.6 (89.0–98.8)
IHA (titer ≥ 1:160)	69.5 (61.2–77.0)	67.6 (60.4–74.2)	100 (96.0–100)

a The assay values were calculated using data from Thai patients in Ubon Ratchathani in northeast Thailand who had melioidosis, Thai healthy donors, and U.S. healthy donors. CI, confidence interval.

The diagnostic sensitivity of ICT using Hcp1 at a concentration of 0.5 mg/ml was higher than those of the Hcp1-ELISA (sensitivity, 89.4% versus 83.0%, *P* = 0.008) and IHA (sensitivity, 69.5%, *P* < 0.001), and the sensitivity of ICT using Hcp1 at a concentration of 1 mg/ml was higher than those of Hcp1-ELISA (sensitivity, 90.1% versus 83.0%, *P* = 0.004) and IHA (sensitivity, 69.5%, *P* < 0.001).

Using Thai donors as a control, the specificity of the Hcp1-ICT using a concentration of 0.5 mg/ml was lower than that of the Hcp1-ELISA (86.2% versus 96.3%, *P* < 0.001) but higher than that of IHA (specificity, 67.6%, *P* < 0.001). The specificity of the Hcp1-ICT at a concentration of 1 mg/ml was lower than that of the Hcp1-ELISA (84.0% versus 96.3%, *P* < 0.001) but higher than that of IHA (specificity, 67.6%, *P* = 0.001).

**FIG 2:**
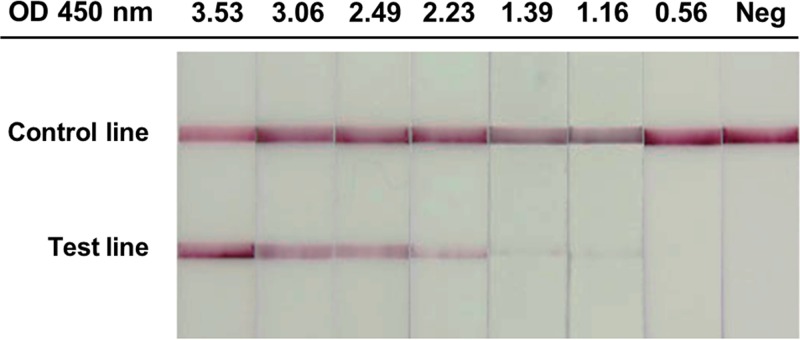
Comparison of results of Hcp1-ICT and Hcp1-ELISA with different OD values.

Using U.S. donors as a control, the specificity of the Hcp1-ICT at concentrations of 0.5 mg/ml and 1 mg/ml was 100%. The specificities of both ICTs were higher than that of the Hcp1-ELISA (100% versus 95.6%, *P* < 0.001) but were not different from that of IHA (100% versus 100%). Kappa analysis of all 419 samples showed that the results of the Hcp1-ICT at concentrations of 0.5 mg/ml and 1 mg/ml were in good agreement with the Hcp1-ELISA results (agreement = 92.4%, kappa = 0.829, and 95% confidence interval [CI] = 0.773 to 0.885 and agreement = 91.2%, kappa = 0.804, and CI = 0.745 to 0.864, respectively) but in moderate agreement with the IHA results (agreement = 79.2%, kappa = 0.555, and 95% CI = 0.473 to 0.638 and agreement = 79.5%, kappa = 0.563, and 95% CI = 0.481 to 0.645, respectively). The lowest OD_450_ value from the Hcp1-ELISA that resulted in a positive Hcp1-ICT was 1.16 (Fig. 2).

### Evaluation of Hcp1-ICT for diagnosis of melioidosis in serum samples.

A diagnostic evaluation of the Hcp1-ICT was further conducted in Udon Thani Hospital, Khon Kaen Hospital, and Nakhon Phanom Hospital in northeast Thailand using serum samples from patients with B. pseudomallei and other infections. Hcp1-ICT at a concentration of 0.5 mg/ml used less antigen for the assay kit assembly and had slightly higher specificity than Hcp1-ICT at a concentration of 1 mg/ml. We thus decided to use Hcp1 at a concentration of 0.5 mg/ml for further evaluation. The Hcp1-ICT results of serum samples from all hospitals, including Sunpasitthiprasong Hospital in Ubon Ratchathani, are summarized in Table 2. The diagnostic sensitivity of the Hcp1-ICT using a total of 487 serum samples from all hospitals in Thailand was 88.3% (CI, 85.1% to 91.0%) and ranged between 80.7% and 91.4%, and the specificity of the total of 207 serum samples from the group of patients with other infections was 91.8% (CI, 87.2% to 95.1%) and ranged between 92.2% and 100%. We previously demonstrated by Hcp1-ELISA that diabetic patients had higher antibody titers for Hcp-1 than nondiabetic patients (19). In this study, we assessed the relationship between diabetic status and the Hcp1-ICT results in 188 melioidosis patients for whom the diabetic status was known. Among these patients, the numbers of the members of the diabetic and nondiabetic groups were 124 (66.0%) and 64 (34.0%), respectively. Hcp1-ICT results were positive for 119/124 (96.0%) of the members of the diabetic group and 54/64 (84.4%) of the members of the nondiabetic group. We also observed a significant association between diabetic status and the results of Hcp1-ICT (*P* = 0.009, Fisher's exact test).

**TABLE 2:** Hcp1-ICT sensitivity, specificity, and 95% confidence interval at a Hcp1 concentration of 0.5 mg/ml^*a*^

Location of serum sample subjects	Subjects with melioidosis		Subjects with other infections		Healthy subjects	
	No. of positive samples/total no. of samples	% sensitivity (CI)	No. of negative samples/total no. of samples	% specificity (CI)	No. of negative samples/total no. of samples	% specificity (CI)
Thailand (total)	430/487	88.3 (85.1–91.0)	193/207	91.8 (87.2–95.1)	174/202	86.1 (80.6–90.6)
Ubon Ratchathani	126/141	89.4 (83.1–93.9)	ND	ND	162/188	86.2 (80.4–90.8)
Udon Thani	181/198	91.4 (86.6–94.9)	48/50	96.0 (86.3–99.5)	12/14	85.7 (57.2–98.2)
Khon Kaen	77/91	84.6 (75.5–91.3)	142/154	92.2 (86.8–95.9)	ND	ND
Nakhon Phanom	46/57	80.7 (68.1–90.0)	3/3	100 (29.2–100)	ND	ND
United States	ND	ND	ND	ND	90/90	100

a The assay values were calculated using samples from Thai patients in four hospitals in northeast Thailand who had melioidosis and other infections and samples from U.S. healthy donors and Thai healthy donors. ND, not done.

The specificity of the total of 202 serum samples from the group consisting of Thai healthy donors was 86.1% (CI, 80.6% to 90.6%) and ranged between 85.7% and 86.2%. The percentage of patients that were culture positive for other organisms that were also Hcp1-ICT positive was 6.8% (14/207). The culture results of these organisms are listed in Table 3. The Hcp1-ICT-positive samples included samples from patients infected with Acinetobacter baumannii (2/21), Bacillus spp. (1/4), Citrobacter koseri (1/2), coagulase test-negative staphylococci (2/20), Corynebacterium spp. (1/5), Candida glabrata (1/1), Escherichia coli (1/15), Klebsiella pneumoniae (2/13), Orientia tsutsugamushi (1/20), and Staphylococcus aureus (2/15). BLASTP searches (https://blast.ncbi.nlm.nih.gov/Blast.cgi) using the protein sequence of rHcp1 were performed against the genomes of these organisms. We did not find any sequences demonstrating significant homology with the rHcp1 in the genomes of these organisms.

**TABLE 3:** Culture results of serum samples positive for Hcp1-ICT

Culture result	Total no. of serum samples	No. (%) of Hcp1-ICT-positive samples
Acinetobacter baumannii	21	2
Aeromonas spp.	1	0
Bacillus spp.	4	1
Burkholderia cepacia	1	0
Citrobacter koseri	2	1
Coagulase-negative staphylococci	20	2
Corynebacterium spp.	5	1
Cryptococcus laurentii	1	0
Cryptococcus neoformans	2	0
Candida albicans	9	0
Candida glabrata	1	1
Candida spp.	1	0
Clostridium perfringens	1	0
Enterococcus faecium	3	0
Escherichia coli	15	1
Elizabethkingia meningoseptica MDR^*a*^	1	0
Enterobacter aerogenes	1	0
Enterococcus faecalis	1	0
Enterococcus spp.	5	0
Haemophilus influenzae	1	0
Klebsiella pneumoniae	13	2
Klebsiella oxytoca	1	0
Leptospira interrogans	20	0
Micrococcus spp.	2	0
Morganella morganii	1	0
Orientia tsutsugamushi	20	1
Pseudomonas aeruginosa	3	0
Pseudomonas spp.	1	0
Streptococcus agalactiae	8	0
Streptococcus pneumoniae	2	0
Streptococcus anginosus	1	0
Streptococcus dysgalactiae	1	0
Streptococcus gordonii	1	0
Streptococcus parasanguinis	1	0
Streptococcus pyogenes	2	0
Group B Streptococcus	1	0
Non-group A, B, or D streptococci	1	0
Staphylococcus aureus	15	2
Viridans streptococci	2	0
Multiple infections	15	0
Total	207	14 (6.8)

a MDR, multidrug resistant.

## DISCUSSION

Melioidosis is a fatal infectious disease that is being increasingly reported worldwide. Statistical modeling recently predicted the occurrence of up to 165,000 human melioidosis cases per year globally and an estimated 89,000 melioidosis deaths (4). Investigators highlighted that underdiagnosis and underreporting of melioidosis are major concerns. Only ∼1,300 cases were reported per year worldwide, which is <1% of the estimated annual incidence (1, 4). Major factors contributing to the low reported incidence are lack of laboratory diagnostic tools and lack of facilities and clinical expertise (1).

Delay in diagnosis could lead to ineffective antimicrobial treatment and mortality. This study focused on the improvement of rapid serodiagnostic tests for melioidosis for the development of a POC test. A prototype lateral flow immunoassay (LFI) for detection of B. pseudomallei capsular polysaccharide in patients' samples has been evaluated, but it provided only 40% sensitivity for blood specimens (14) The ICT developed in this study detected IgG antibody to Hcp1. Hcp1 is an integral component of the T6SS apparatus, which is highly expressed only following infection of a host (20, 21). Our data suggest that the Hcp1-ICT developed here is a reliable and sensitive serological test that achieved 88.3% sensitivity and 86.1% specificity for Thai healthy donors, 100% specificity for U.S. healthy donors, and 91.8% specificity for patients with other infections. The results of the Hcp1-ICT were consistent with the Hcp1-ELISA results (19) as demonstrated by the high agreement between these two assays. The lower specificity observed in the Hcp1-ICT than in the Hcp1-ELISA in the Thai donor group and the higher sensitivity observed in the Hcp1-ICT than in the Hcp1-ELISA in the Thai melioidosis group (Table 1) may be associated with one or more of the following factors: (i) the use of different serum dilutions (1:2,000 for Hcp1-ELISA versus 1:13 for Hcp1-ICT); (ii) the use of different amplification signals (horseradish peroxidase reaction versus colloidal gold reaction); and (iii) the use of different methods for reading results (the presence of reddish lines judged by the naked eye versus OD values above a cutoff value determined by the use of a microplate reader).

Our observations, however, indicate that at least 10% of patients did not produce antibody to Hcp1, resulting in a negative result with both the Hcp1-ICT and Hcp1-ELISA. The negative result may have been due to the immunocompromised status of the patients or to the time of diagnosis having been too early to detect a rising IgG level. Our previous studies determined antibodies to OPS antigen and demonstrated that sera from most of the patients in the nonreacting group also gave negative results for antibody to OPS. Lim et al. demonstrated that melioidosis patient sera have a significantly highly titer of Hcp1-specific antibodies for both IgG and IgM than sera from healthy controls (22). It is unknown whether detection of IgM against the Hcp1 would improve the sensitivity of the method. Further investigations are required to address these issues.

Although the specificity of ICT was high, it was not 100% in an area where infection is endemic such as Thailand. The proportion of patients that were culture positive for other organisms and were Hcp1-ICT positive was 6.8%. In this study, we did not have information regarding the time points at which the specimens were taken for these patients. Some patients who were admitted to a hospital for a long period may have received antibiotic treatment, and culture results were negative for B. pseudomallei for those patients. It is possible that they subsequently acquired other antibiotic-resistant bacterial infections during treatment. This type of information will be useful to know for the future evaluation of the Hcp1-ICT assay.

It is also unclear why the seropositivity of Thai healthy donors was higher than that of patients with other infections. The background serological positivity rate in the healthy subjects in northeast Thailand was found to be 14% with Hcp1-ICT, which may have been a result of repeated exposures to B. pseudomallei or to closely related Burkholderia species (1, 23). Therefore, culture is still required to confirm the presence of B. pseudomallei as well as for drug susceptibility testing.

Delays in diagnosis could lead to ineffective antimicrobial treatment, morbidity, and mortality. Although hospital laboratories remain a centralized laboratory for culture methods, Hcp1-ICT would be a first-line test with a short turnaround time. Using the same serum samples, our data suggest that the Hcp1-ICT outperformed the current serological IHA (15). The advantage of the Hcp1-ICT is that it is standardized, rapid, and simple. It could be a point-of-care test that does not require equipment, specialized facilities, or extensive training. The results of Hcp1-ICT form permanent lines on a membrane that are stable and can be read at any convenient time. The Hcp1-ICT may be used to replace the IHA for screening for the presence of antibodies, but the Hcp1-ICT cannot determine antibody titers. At present, when produced on a small scale for research purposes, the cost of Hcp1-ICT is about 8 to 10 U.S. dollars per assembled test, which could be reduced by producing the tests on a large scale or by further developing it using less-expensive materials. The test is stable at room temperature for at least 1 year. On the basis of our findings, we conclude that the Hcp1-ICT is a potential POC test which may be a rapid, reliable screening tool for serological surveillance and clinical diagnosis of melioidosis.
